# Encoding the states of interacting proteins to facilitate biological pathways reconstruction

**DOI:** 10.1186/1745-6150-5-52

**Published:** 2010-08-13

**Authors:** Alberto Termanini, Paolo Tieri, Claudio Franceschi

**Affiliations:** 1 'L. Galvani' Interdepartmental Center, University of Bologna, Via San Giacomo 12, 40126 Bologna, Italy; 2 Department of Experimental Pathology, University of Bologna, Via San Giacomo 12, 40126 Bologna, Italy

## Abstract

**Background:**

In a systems biology perspective, protein-protein interactions (PPI) are encoded in machine-readable formats to avoid issues encountered in their retrieval for the reconstruction of comprehensive interaction maps and biological pathways. However, the information stored in electronic formats currently used doesn't allow a valid automatic reconstruction of biological pathways.

**Results:**

We propose a logical model of PPI that takes into account the "state" of proteins before and after the interaction. This information is necessary for proper reconstruction of the pathway.

**Conclusions:**

The adoption of the proposed model, which can be easily integrated into existing machine-readable formats used to store the PPI data, would facilitate the automatic or semi-automated reconstruction of biological pathways.

**Reviewers:**

This article was reviewed by Dr. Wen-Yu Chung (nominated by Kateryna Makova), Dr. Carl Herrmann (nominated by Dr. Purificación López-García) and Dr. Arcady Mushegian.

## Background

The reconstruction of biological systems for computational analyses relies on the existence of data describing components as well as their interactions and relationships [1,2]. It is well established that understanding the essence of protein interactions is a key factor for the development of systems biology as well as novel therapeutics [3].

Nevertheless, it is also well recognized the difficulty of collecting such kind of information through scientific literature, especially when the number of considered proteins is large, because scientific literature contains PPI data in the unstructured format of human natural language. For this reason, a subset of protein interactions gets into PPI databases such as BIND [4], BioGRID [5], DIP [6], HPRD [7], IntAct [8], MINT [9] and MIPS [10], allowing for fast retrieval and computational analysis of large datasets [11,12]. In this way the reconstruction of biological systems can be obtained by parsing "formal languages" instead of natural ones, making the identification and the retrieval of such very valuable information easier, faster, and much more reliable [13]. However, for pathways reconstruction the interaction data, intended as a simple "binary" relationship between two elements belonging to the pathways, are not sufficient. In particular, it is essential to know the direction of the binary relations and the conditions in which they occurs. For example, a PPI could be achieved only if one of these proteins appears to be phosphorylated at certain residues.

In order to store this useful kind of information, we focus on the adoption of a general logical model to make PPI data immediately usable for automatic or semi-automated reconstruction of biological pathways, which can be easily integrated into existing electronic formats used to store the PPI data such as PSI-MI [14] and BioPax [15].

## Results and Discussion

### A logical model of PPI that considers the states of the interacting proteins

The general, purely descriptive model we propose takes into consideration the protein "state" before and after the interaction, and can be integrated, adapted or adopted in already existing standards for PPI interaction data.

With "state" of a protein we mean all the information that can describe the protein properties, in particular those that are significant for the interaction and for the protein functions. For example, if a protein will change its conformation, its function or its cellular localization after the interaction, this information should be recorded. We want to stress that we are interested in that kind of changes (or its absence) in the interacting protein also before the interaction. In this way, we can define under what conditions will happen the interaction (conformation, post-translational modification such as phosphorylated residues, sub-cellular localization etc.).

Although these data are not necessary in systems biology approaches that want to analyze the network of interactions in order to study its topological properties and for identify functional modules and motifs, these are essential for pathways reconstruction.

**Figure 1:**
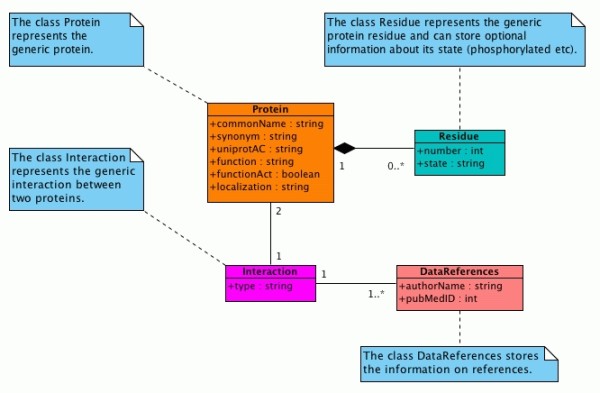
**Class diagram of the protein-protein interaction model**.

**Figure 2:**
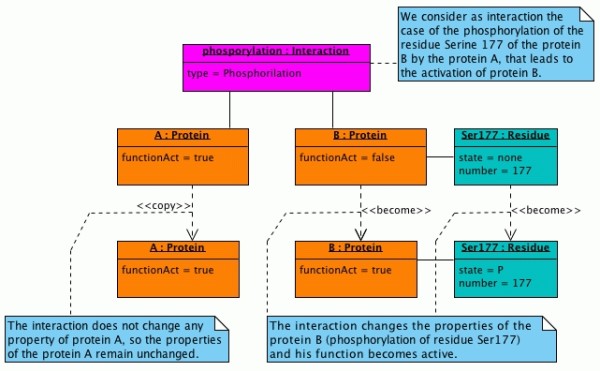
**Object Diagram of an example of interaction**.

UML [16] is a language for modeling software or non-software systems and has been proven successful in the modeling of large and complex systems. UML uses graphical notations to express the design of the projects. We describe our model of PPI interaction with an UML Class Diagram (Fig. 1) and we show an example with an UML Object Diagram (Fig. 2).

Classes and objects are concepts derived from the Object Oriented Programming (OOP), a programming paradigm that uses "objects" and their interactions to design applications and computer programs.

A class defines the characteristics and behaviours of a thing. For example, the class Car defines the possible characteristics and behaviours common to, and shared by all cars such as the type of engine, the shape of the body and the maximum number of passengers.

An object is an instance of a class. Then, for example, we can have different objects of type Car, and each object represents a particular automobile having certain defined characteristics.

### Class Diagram of the model

In UML, a Class Diagram gives an overview of the system by showing its classes and the relationships among them. The relationships between classes are shown as connecting links. Class diagrams are static diagrams because they display the elements that are interacting, but not what it is happening when the interactions occur. Class names begin with a capital letter.

In our model (Fig. 1), the interaction between two proteins is described by specifying the "state" of the two proteins before and after the interaction. At this experimental stage we choose to define the classes Protein (Table 1), Residue (Table 2), Interaction (Table 3), DataReferences (Table 4) and the relationships among them.

**Table 1:** Attributes related to the class Protein.

Name	Type	Multiplicity	Description
*commonName*	string	1	The common name of the protein.
*synonym*	string	0..*	Other names of the protein.
*uniprotAC*	string	1..*	The Accession Number (AC) from the UniProt Database.
*Function*	string	0..*	A function of the protein.
*functionAct*	boolean	0..*	True if the function described in the field 'function' is active, false otherwise.
*localization*	string	1..*	One or more protein cellular localization.

**Table 2:** Attributes related to the class Residue.

Name	Type	Multiplicity	Description
Number	numeric	0..*	The number of the residue of the protein.
State	char	1..*	The state of the residue (for example 'P' if phosphorylated, 'U' if ubiquitinated etc.).

**Table 3:** Attributes related to the class Interaction.

Name	Type	Multiplicity	Description
Type	string	1	The type of the interaction (ubiquitination etc.).

**Table 4:** Attributes related to the class DataReferences.

Name	Type	Multiplicity	Description
authorName	string	1..*	The names of the authors of the experiment that describe the interaction data.
pubMedID	string	1	The Pub Med ID (PMID) of the paper in which the interaction is described.

### Class Protein

The class Protein represents the generic protein. Note that between the class Protein and the class Residue there is a "composition" relationship. In UML, a composition relationship is used when an object is made up of other objects and the whole and parts have coincident lifetimes.

The multiplicity of the relationship is 1 for Protein and (0..*) for Residue. This means that there can be zero, one or more Residue for each Protein. In fact, we don't need to store the information of all the residues of the protein because we would like to define the interaction between two proteins and not the exact residue sequence.

Thus, for example, if we know that the interaction takes place only if a certain residue is phosphorylated, it is enough to consider only the information about this residue and its state (phosphorylated).

### Class Residue

The class Residue represents the generic residue of the protein. There is a "composition" relationship between Residue and Protein.

### Class Interaction

The class Interaction represents the generic interaction between two proteins. Note that between the class Protein and the class Interaction there is an "association" relationship. In UML, an association relationship is used when a class must be necessarily associated to another class.

The multiplicity of the relationship is 1 for Interaction and 2 for Protein. This indicates that for every object of type Interaction there must be two objects of type Protein. This is because we want to model the interaction that takes place between two proteins.

### Class DataReferences

The class DataReferences stores the information on the article reference where the interaction is described.

### An example of interaction

We show an example of interaction in the Object Diagram in Fig. 2. In UML, a pictorial representation of the instances of classes (i.e. objects) and the relationships among them is called "Object Diagram". It looks similar to a Class Diagram and uses similar notations to denote relationships. The object names are separated from the class names by a ":" and are underlined.

In the example we consider as interaction the case of the phosphorylation of the residue "Ser177" of a protein "B" by the protein "A", that leads to the activation for a given function of the protein "B".

It is important to say that we store the "state" of both proteins before and after the interaction. In the example, for the protein "A" we have an indicator "copy" of the object "A" because the interaction doesn't change any of the properties (considered by the model) of the protein "A". Finally, we have an indicator "become" for the objects "B" and "Ser177" because the properties of these two objects are changed after the interaction.

This example also shows how the model is able to take into account the "direction", simply defined as the direction of the arrow in the binary relation between A and B. In fact, it is clear that the direction is from A to B because is A that change the state of B. During the reconstruction of a hypothetical pathway in which are involved the proteins A and B, starting from these data we could say that the protein A, in its native conformation, can acts on the protein B when it is in its native conformation, and that the result is the activation of the function of the protein B.

### Model expansion

The proposed model was kept purposely simple to easily transmit the idea that adopting a model that takes into account the "state" of interacting proteins is important for pathways reconstruction. However, as the UML can handle even highly complex models, it can be easily expanded to consider other information. In general, a model may be more or less complex depending on many factors and in accordance with the purpose to be achieved by the use of the model.

For example, we may want to add in the "state" of the interacting proteins the knowledge of the conformations assumed by the proteins. In fact, the amino-acid sequence of a protein determines its native conformation, which in turn determines its biological function. In our case, since the aim of the model is to facilitate the reconstruction of biological pathways from PPI data, and not to perform complex simulations like molecular docking, a simple solution might be to insert a variable into the class Protein to store the type of the assumed conformation (α = native; β1 = alternative conformation n.1; β1 = alternative conformation n.2; and so on).

Another more complex way would be adding the variables dX, dY and dZ in class Residue. In the native conformation the variables dX, dY and dZ are set to zero for all residues. In a given alternative conformation, for the i-th residue, the variables dXi, dYi and dZi contains the distance from the central point of the residue in the native conformation respectively along the axis X, Y, Z. Gradually complicating the model, we can get up to consider the coordinates of each atom of the protein, but of course this data is rarely available and, in addition, it would be excessive for the purpose of the model.

### Adoption of the model and pathway reconstruction

As shown in the example above, the model takes into account the "state" of interacting proteins before and after the interaction. Thanks to this, the model is able to provide necessary information to reconstruct the pathway from the PPI data. Instead, as described above, the only binary information of the type "protein A interacts with protein B" is not sufficient.

Finally, the data of the previous example may be presented in a series of XML code lines although, as mentioned above, these information could be easily integrated into currently adopted formats used to encode PPI data instead of creating new.

It is important to emphasize that the information considered by the model should be included within the PPI data. Although current standards are available for modeling of biological pathways such as SBML [17] and mEPN [18], we don't want to place our model at the level of those languages. In fact, the problem we faced is located at the previous step, namely the reconstruction of pathways from the "raw" data present in PPI databases.

### Development of the model

We propose that this project will be developed by the community as an open-software project. The project web site is accessible at the following URL: http://bioinfo.homeunix.org/forum/

## Conclusions

Encoded protein-protein interactions in a machine-readable format avoid waste of time and uncertainties, difficulties and typical issues encountered in the retrieval of such data and in the reconstruction of comprehensive interaction maps and biological pathways based on interaction data.

However, the knowledge of only the "binary" interactions between the elements belonging to a pathway is not sufficient to its reconstruction. For example, it is necessary to know the direction of the relationship and the conditions under which it occurs.

Our simple logical model of PPI shows how to take account of this essential information considering the "state" of interacting proteins before and after the interaction. The lack of these data (that would be saved in machine-readable standards adopting this model) translates into a long work by the researchers that are forced to manually search in published papers. This model can be easily extended to describe the interactions between proteins and small molecules, between proteins and protein complexes etc. and can be integrated into the existing standards encoding protein interactions. Its adoption will allow for an automatic or semi-automatic reconstruction of biological pathways from the interaction data, decreasing the probability of making errors and reducing the time required for this task.

## Methods

### UML diagrams

The UML diagrams were created with the design tool Visual Paradigm for UML v7.2 [19] (Visual Paradigm International, Hong Kong).

## Competing interests

The authors declare that they have no competing interests.

## Authors' contributions

AT and PT conceived the experimental proposal, participated in its design and coordination and contributed to write the manuscript. CF participated in its design and coordination and contributed to write the manuscript. All authors read and approved the final manuscript.

## Reviewers' comments

### Reviewer's report 1

*Wen-Yu Chung, Center for Systems Biology, Department of Molecular and Cell Biology, The University of Texas at Dallas, Richardson, Texas, USA (nominated by Kateryna Makova, Department of Biology, The Pennsylvania State University University Park, Pennsylvania, USA)*.

Main comments

1) The proposed work described a logic model that collects critical information for protein-protein interactions. The authors stated this method would be able to assist reconstructing biological pathways. The idea of logic model fits well with systems biology. One can model the system using elements such as input, output and activities. The simple model authors proposed is able to save residue information that is essential for the interaction.

2) The classes and attributes described in the manuscript did not capture the complexity of protein interactions. For example, the structure conformation is not included. But, the authors presented that an extension could be done by inserting variables in the class Protein, so the knowledge of conformation changes can be preserved. Other more complex scenarios can be added in similar ways in the future to obtain a comprehensive model.

3) The authors emphasized that the logical model is different from Systems Biology Markup Language (SBML), such that their method focused on the first step of building pathways, naming retrieving "raw" data from PPI databases. In this aspect, the two approaches could potentially accompany each other since first they targeted at different steps of pathway construction, and second both employ XML in practice.

Minor issues not for publication

1) In "Results and Discussion", at "Adoption of the model and pathway reconstruction", the last paragraph, the citation (url link, ref.[17]) for SBML is http://sbml.org/.

#### Author's response

*We have corrected the URL*.

2) Please indicate in the manuscript whether a more detailed manual or examples will be available, and if so, where.

#### Author's response

*We have added the link to the project website*.

### Reviewer's report 2

*Carl Herrmann, TAGC & Université de la Méditerranée (Aix-Marseille II), Marseille, France (nominated by Purificación López-García, Unité d'Ecologie, Systématique et Evolution, Université Paris-Sud 11, Orsay, France)*.

In this article, the authors present a logical model of protein-protein interactions that allows to take into account the modifications induced by the interaction at certain residues. This information is usually not available in common PPI databases such as Intact or BIND. The model is based on the UML modeling language, and they present a Class diagram representing the various classes defined. This approach is interesting, as, as emphasized in the introduction, most description of such processes are available in the literature in natural language, which makes its systematic use difficult for bioinformatics approaches. Hence, any attempt to introduce a systematic nomenclature or description of biological processes is welcome.

My first comment is that studies analyzing interactomes are not necessarily aiming at reconstructing pathways. Functional modules extracted from PPI data could represent e.g. protein complexes acting as a cellular machinery. In this case, the exact nature of the interaction (i.e. which residue is involved, the presence of modifications, etc...) are usually not needed. Therefore, the information currently available in PPI databases is sufficient for many studies.

#### Author's response

*We partially agree. Although this information is not always necessary, as in systems biology approaches in which you want to look at the topology of the network of interactions for properties of the network and for identify functional modules and motifs, there are cases where the lack of information that would be saved from machine-readable standards adopting this model translates into a long work by the researchers that are forced to find it manually from published papers. This model is designed to improve the existing standards so that you can use this kind of information in case they are needed, such as in the case of the reconstruction of pathways*.

The authors mention other projects in the introduction which go into similar directions: they cite SBGN, BioPAX, and HUPO-PSI, but one very important project in this field is not mentioned: SBML.

#### Author's response

*We have changed the manuscript accordingly*.

However, general interaction/pathway databases such as Reactome use the SBML representation for "reactions", which is grounded on UML. It seems to me that SBML already allows for the introduction of informations such as the modification of an entity, e.g. the phosphorylation of a protein in a reaction. In particular, the Systems Biology Ontology (SBO), which is closely integrated into SBML, contains an "interaction" branch, which contains terms such as phosphorylation (SBO:0000216), a sub-term of the "Addition of chemical group" term (SBO:0000210). Also, the PSI-MOD ontology contains terms such as "phosphorylated residue" (MOD:00696). Hence, my concern is that the model presented by the authors might be redundant with already existing representations or models, as those cited above. If this is not the case, the authors should clearly explain the difference of their approach with respect to existing ones, and make clear why it does not fit into e.g. SBML type descriptions. Indeed, the danger is that multiple independent initiatives will fragment the field instead of converging to a common, unified, description scheme.

#### Author's response

*We don't agree: we are considering the previous step. That is when, starting from protein-interaction data present in PPI database, you want to reconstruct a pathway. It is at this step that that information that could be memorized by the implementation of the proposed model becomes essential. After that, once the pathway has been reconstructed, it can be stored in appropriate formats such as the Systems Biology Markup Language (SBML), the modified Edinburgh Pathway Notation (mEPN) and so on*.

I acknowledge that the authors are aware of this, as they explain that "The general, purely descriptive model we propose [...] can be integrated, adapted or adopted in already existing standards such as the PSI-MI" (first paragraph of the discussion) or "[...] these information could be easily integrated into currently adopted formats used to encode PPI data instead of creating new." (last paragraph of the discussion). I would appreciate if the authors would make these statements more precise, and give details about the possible integration of their approach into existing projects.

#### Author's response

*As previously said, we don't want to insert our model at the level of the languages used for simulation and modeling of biological pathways. We want to place our model at the previous level, when you need to reconstruct a pathway starting from the data available in PPI databases. The models used in these databases could integrate the concept of "state" of interacting proteins before and after the interaction to present this valuable information in electronic format*.

### Reviewer's report 3

*Arcady Mushegian, Department of Binformatics, Stowers Institute for Medical Research, Kansas City, Missouri, USA*.

I have very little to say about this manuscript, not being in expert in automatic text processing and relational modeling. I expect that improvement in machine-readability of the data on protein interactions will be useful for more accurate reconstruction of the pathways, but I do not see that this study has shown how this approach makes this actually happen in any specific case.

#### Author's response

*Currently, to our knowledge, there is no model that takes into account the "state" of interacting proteins before and after the interaction. Consequently, there are not databases where this kind of information is present in machine-readable format, and reconstruction of pathways thus requires more work as this information should be sought directly in the papers. Being a logical model, it is natural that does not resolve a particular case, but its adoption and implementation in one or more of the standard electronic formats protein interaction would allow other researchers to solve in less time with fewer errors and an unlimited number of special cases of interest*.
